# Essay

**Published:** 1857-04

**Authors:** Thomas S. Denny


### ARTICLE III.

*Essay*. By THOS. S. DENNY, M. D., read before the Atlanta Medical Society, and published by that body.

*Gentlemen of the Society*:—It has been in the midst of many embarrassments, that I have been compelled to prepare an Essay to be read before your body. My humble effort will, I trust, be treated with leniency.

To adopt Dr. Wilson's theory, contained in an Essay published in our Journal for May, 1856, we would be doing ourselves a great injustice; burdening ourselves with much more responsibility than we ought, rightfully to assume.

The number of Empirics in the Profession, i. e., of those who became so after being clothed with the privileges attached to graduation, is very small, compared with the number who are permitted by legal authority, or rather, not restrained by legal enactments, from putting forth their nostrums, who never even read a page of medicine in their lives, though our Profession is now in the very act of providing a remedy for the former evil, by requiring the expulsion from our ranks, of those who will violate our yet imperfect code. The great success to be attributed to Empirics, in the spread of their so called *remedies*, and of their consequent accumulation of wealth, is to be explained by the credulity of human nature.

Dr. Brown, in his "Observations," says, it is the bold, unscrupulous assertion of infallibility of the Charlatan, which secures his success.

With reference to his theory of incompetency, I may be permitted to say that Colleges, and not the irregular members of the Profession, or of Empirics, proper, are the best judges of competency, and the less we have to do, with the latter especially, the better. I, for one, am willing to leave them to their notoriety.

It is the public mind which we must endeavor to enlighten, but only to such a degree as to show the vast superiority of the regular medical profession, over the miserable pretenders who infest the world by thousands, for the sole purpose of wresting from the ignorant the hard earned reward of their industry, and even from the wealthy and frequently equally ignorant class, the sanction of their influence to their artfully contrived impositions.

But, another subject, of, I trust, more interest, I will endeavor to illustrate, at least according to my own humble view of it, the nature of disease in the same organ.

I cannot regard disease as so infinitely diversified in its nature as many of our profession are disposed to think. One of the collateral arguments I would advance in support of the opinion, is the utter discontinuance of such a host of remedial agents, as were once regarded as specifics in disease, and indispensable to its eradication. We know now that Hippocrates, Galen, even Cullen—may I not say Rush—would, could they have lived to the present era, be ready to ridicule the use of many of their favorite remedies, and would smile upon our efforts to advance the science of medicine by its simplification.

Phthisis Pulmonalis, Consumption proper, is only marked being accompanied, or not, by biliary derangement. Its variations otherwise, are very few. There is no essential difference in fevers, it is only in the seat of local affection, and in its manifestations, that they are distinguished. "Fire," says Dr. Rush, "is a unit, whether produced by heat, friction, electricity, or any other cause." The treatment although varied, does not very materially differ; it is not more than subject to the general principles of medicine.

The theory that there is a great identity in the nature of disease, (at least in diseases of the same organ,) is not very ancient, for even the great, but unpractical Dr. Good has been, and is still, more and more criticized, and his "class, order, genius, species, and variety" of disease, is read, as Dr. Samuel Johnson declares he "had read Miltons Paradise Lost, more as a duty than a pleasure."

The French School of Medicine has had a great deal to do with the perpetuation of *variety* of disease, in its peculiar sense, and has so multiplied the distinctions, that the real differences have become somewhat obscured. The ideas of that class of Theorists and Practitioners have become confused, in matters of science as well as politics, and both they, and consequently we, are frequently at a loss to embrace their ideas. I am willing to award to them great earnestness, and even more than they claim, great enthusiasm, but most respectfully decline to give any but a very limited sanction to many of their views. Indeed, the necessary retractions, which their followers, (imitators at least,) are frequently compelled to make, is evidence sufficient to point out that imperfection is attached to much that they claim as original, and especially adapted to disease.

The idea which I wish to elucidate is, that disorders of the same organs are, from the very nature of things, necessarily similar in kind, though different in degree. Inflammation of the liver is attended with a suppression of the secretion, natural to that organ, and a total want of action of the same viscus is accompanied by the same condition; a total suppression of the secretion, natural to any of the internal secretory organs, is frequently, the immediate precursor of the higher state of inflammation, and more—the same remedies are employed to restore the natural action of the organ in both cases.

Analogy is pertinent to our subject. Does it seem natural that the human family should, for centuries, be subject to suffering, while those whose peculiar office it is to afford relief, are groping in the dark, after imagined differences, narrowed down to a hair's breadth, into infinite distinctions, in diseases of the same organ. The food of man is divided into two *great* classes, and his health is affected but by a limited variation in temperature of the atmosphere. Certain classes of disease prevail *principally* in certain portions of the globe; and although they are somewhat modified in intensity, their peculiar nature is the same.

Take the diseases of hot and cold climates, do they not affect different organs respectively, subject only to the peculiarities of the constitution of the individual? Consumption is generally regarded as aggravated and fatal in cold latitudes, and soothed in warm, southern climes. Bilious affections, and cutaneous eruptions prevail in warm regions—the first from direct action of heat, the latter from impurities of the blood, caused by inappropriate dietetic indulgences.

In the language of an original, chaste, and thoughtful writer,* upon a subject of a kindred nature, "There is certainly an original difference in the conditions of men, and of

* Gregory's Comparative Views, p. 123.

nations, but this is not so great as at first view it seems to be. Human nature consists of the same principles every where. In some people, one principle is naturally stronger than it is in others, but exercise and proper culture will do much to supply the deficiency. The inhabitants of cold climates having less natural warmth, and sensibility of heat, enter but very faintly into those refinements of the social principle, in which men of a different temper delight. But, if such refinements are capable of affording to the mind innocent and substantial pleasure, it should be the business of Philosophy to search into the proper methods of cultivating and improving them."

It will readily be perceived that the objections which we have, are against the infinite modifications of the same disease, which are attempted to be elaborated, and refined, until imagination is unable to follow the intricate labyrinth, and becomes confused in following chimeras which would require such an unlimited number of remedial agents, as to exceed the bounds of the Materia Medica, and has doubtless driven many to the one remedy system—one cure for all diseases—the very essence of Empiricism.

Professor Dickson, in his Elements of Medicine, speaking of *classes* of fever, says: "I do not object to the nicest diagnosis, the drawing of the most delicate lines of distinctions. I applaud rather all attempts in this way, and study with pleasure, as well as profit, the Essays of Copland, Gerhard, Jackson, Ware and Jenner. But, even in these ingenious and useful efforts at analysis, these vivid deliniations of differences, we are perpetually presented with striking analogies, striking resemblances, the intrusion of characteristic symptoms, where they were not looked for, and their absence, where they were confidently expected. The Synocha and Synochus, the typhus and typhoid, run together, inexplicably. Partisan advocates of special doctrines often find no reply available, but a denial of some alledged fact as a suggestion either of error, or carelessness in observation. The impartial judge, the earnest student, is not satisfied, however, with this mode of conducting the investigation; and in summing up for the purpose of arriving at the truth, feels himself under the necessity of regarding with equal eye, and weighing with the same scales the statements on both sides of the question, accepting no 'foregone conclusion.'"

In our humble opinion, it would require but a few more years of life to the above candid author, to satisfy him as to the still greater analogy of fevers in general. He is now in the position of the celebrated Dr. Watts, who, not long before his death, gave up the divinity of the third person in the Godhead, as preparatory to his unconditional acceptance of Unitarianism; but whose life was not spared to make the acknowledgment.

That there is a judicious discrimination to be made between all forms of disease, we know, by daily experience, but it is certainly true, also, that imagination has been suffered to run riot over the field, precisely on account of the difficulty of making real distinctions, and satisfying the mind as to the exact nature of the disorder.

The introductory remarks of Dr. Paris to his Pharmacologia, familiar to every student of medicine, are also very appropriate to our present subject; but the refining process is adopted even by him, and carried to an excess, with intended illustrations, frequently devoid of applicability.

A reference to the American Journal of Medical Sciences for July, 1856, page 149, will still farther illustrate and confirm our views on this subject, and I shall consider myself more than fortunate, if I shall have been the means of causing one more thought upon a subject which is forcing itself daily more and more, upon our notice.

